# COVID-19 Delta Variant: Perceptions, Worries, and Vaccine-Booster Acceptability among Healthcare Workers

**DOI:** 10.3390/healthcare9111566

**Published:** 2021-11-17

**Authors:** Khalid Alhasan, Fadi Aljamaan, Mohamad-Hani Temsah, Fatimah Alshahrani, Rolan Bassrawi, Ali Alhaboob, Rasha Assiri, Shuliweeh Alenezi, Ali Alaraj, Reham I. Alhomoudi, Mohammed A. Batais, Lama Al-Eyadhy, Rabih Halwani, Naif AbdulMajeed, Ahmed Al-Jedai, Abdulrahman Senjab, Ziad A. Memish, Sarah Al-Subaie, Mazin Barry, Jaffar A. Al-Tawfiq

**Affiliations:** 1 Pediatric Department, College of Medicine, King Saud University, Riyadh 11451, Saudi Arabia; kalhasan@ksu.edu.sa (K.A.); drhbooob@gmail.com (A.A.); Lama.Aleyadhy@gmail.com (L.A.-E.); salsubaie@ksu.edu.sa (S.A.-S.); 2 Pediatric Department, King Saud University Medical City, Riyadh 11362, Saudi Arabia; rkbassrawi@ksu.edu.sa (R.B.); Reham913@gmail.com (R.I.A.); Dr.naifq@gmail.com (N.A.); 3 Critical Care Department, College of Medicine, King Saud University, Riyadh 11451, Saudi Arabia; faljamaan@ksu.edu.sa; 4 Prince Abdullah Ben Khaled Celiac Disease Research Chair, Department of Pediatrics, Faculty of Medicine, King Saud University, Riyadh 11451, Saudi Arabia; 5 Division of Infectious Diseases, Department of Internal Medicine, College of Medicine, King Saud University Medical City, King Saud University, Riyadh 11451, Saudi Arabia; falshahrani1@ksu.edu.sa (F.A.); mbarry@ksu.edu.sa (M.B.); 6 Department of Basic Medical Sciences, College of Medicine, Princess Nourah Bent Abdulrahman University, Riyadh 11451, Saudi Arabia; raassiri@pnu.edu.sa; 7 Department of Psychiatry, College of Medicine, King Saud University, Riyadh 11451, Saudi Arabia; salenizi@ksu.edu.sa; 8 Department of Medicine, College of Medicine, Qassim University, Buraydah 51452, Saudi Arabia; al_araj@hotmail.com; 9 Department of Medicine, Doctor Sulaiman Al Habib Medical Group, Riyadh 11643, Saudi Arabia; 10 Department of Family and Community Medicine, College of Medicine, King Saud University, Riyadh 11451, Saudi Arabia; drmohammed34@gmail.com; 11 Sharjah Institute of Medical Research, University of Sharjah, Sharjah 27272, United Arab Emirates; rhalwani@sharjah.ac.ae; 12 Department of Clinical Sciences, College of Medicine, University of Sharjah, Sharjah 27272, United Arab Emirates; 13 Pediatric Nephrology Department, Prince Sultan Military Medical City, Riyadh 12233, Saudi Arabia; 14 Deputyship of Therapeutic Affairs, Ministry of Health, Riyadh 11176, Saudi Arabia; ahaljedai@moh.gov.sa; 15 Colleges of Medicine, Alfaisal University, Riyadh 11533, Saudi Arabia; asenjab@alfaisal.edu; 16 King Saud Medical City, Ministry of Health & Alfaisal University, Riyadh 11533, Saudi Arabia; zmemish@yahoo.com; 17 Hubert Department of Global Health, Emory University, Atlanta, GA 30322, USA; 18 Specialty Internal Medicine and Quality Department, Johns Hopkins Aramco Healthcare, Dhahran 34465, Saudi Arabia; jaltawfi@yahoo.com; 19 Department of Medicine, Infectious Disease Division, Indiana University School of Medicine, Indianapolis, IN 46202, USA; 20 Department of Medicine, Infectious Disease Division, Johns Hopkins University School of Medicine, Baltimore, MD 21205, USA

**Keywords:** COVID-19 vaccine booster, COVID-19 delta variant, healthcare workers’ perceptions, travel worry

## Abstract

**Background:** As the COVID-19 Delta variant has spread across the globe, healthcare workers’ (HCWs) knowledge, worries, and vaccine booster acceptance should be assessed. **Methods:** Online questionnaires aimed at HCWs in Saudi Arabia were distributed between 9 and 12 August 2021, aiming to evaluate HCWs’ perceptions and worries about the Delta variant as well as their feelings about receiving a booster-vaccine. **Results:** A total of 1279 HCWs participated, with 51.1% being physicians and 41.7% nurses. 92.5% were aware of the emergence of the Delta variant. Still, only 28.7% were found to have sufficient knowledge of the variant, and their level of worry about it was higher than their level of worry about the Alpha variant (2.32/5 versus 1.79/5). The main information sources cited by the participants were social media (50.5%), while 30.5% used scientific journals. Overall, 55.3% were willing to receive a vaccine booster, while one third would have preferred to receive a new mRNA vaccine specifically developed for the Delta variant. Factors associated with vaccine booster acceptance were receiving both vaccination doses (*p* = 0.008), believing that the Pfizer-BioNTech BNT162b2 vaccine is effective against variants (*p* < 0.001), and agreement that mixing/matching vaccines is effective against variants (*p* < 0.001). **Conclusions:** A high percentage of HCWs were aware of the Delta variant, but only a small fraction had decent quality of knowledge about it. The participants exhibited high worry levels and showed a modest acceptance of receiving a vaccine booster dose. These results should encourage public health officials to scale up educational efforts to disseminate reliable information about the different variants and provide recommendations about receiving a vaccine booster. Further research on methods to alleviate HCWs’ worries about emerging variants is warranted.

## 1. Introduction

As the coronavirus disease 2019 (COVID-19) pandemic became a major global health crisis [1], evidence that Severe Acute Respiratory Syndrome Coronavirus 2 (SARS-CoV-2) could mutate over time mounted; at present, multiple SARS-CoV-2 variants are circulating globally [2]. India has experienced a surge in cases of COVID-19 since late March 2021, with more than 400,000 cases and 4000 deaths reported each day in early May 2021 [1]. Since then, the SARS-CoV-2 Delta variant, known as B.1.617, which was first reported in India late last year, has spread to more than 100 countries—including the United States, Singapore, and the United Kingdom [3]. Researchers have since found three subtypes, known as B.1.617.1 (the ‘original’ B.1.617), B.1.617.2, and B.1.617.3, each of which has a slightly different genetic make-up [3].

The Delta variant is characterized by the spike protein mutations T19R, Δ157–158, L452R, T478K, D614G, P681R, and D950N; several of these mutations may affect immune responses directed toward the key antigenic regions of the receptor-binding protein (RBD L452R and T478K mutations) and the deletion of part of the N-terminal domain. In addition, P681R can be found at the S1–S2 cleavage site, and it appears that strains with mutations at this site may have increased rates of replication, leading to higher viral loads and increased levels of transmission [3].

The Delta variant of SARS-CoV-2 initially detected in India in September 2020 and had spread subsequently to 115 countries globally [4]. The emergence of the Delta variant in India was followed by a significant increase in the cases and in the positivity rate to reach 30% at the end of April 2021 [5]. In Delhi, the Delta variant accounted for 60% of all sequenced samples [5,6]. In Saudi Arabia, a recent pre-publication study of 320 SARS-CoV-2 sequenced samples showed that 40.9% to be Delta variant, 15.9% Beta, and 11.6% alpha variant [7]. Neutralizing antibodies collected 12 months after infection from convalescent sera were fourfold less potent against the Delta variant compared to the Alpha variant [8]. The estimated reproductive number of the Delta variant was 5.08 compared to the 2.79 for the ancestral strain [9]. Investigation of an outbreak in the gym showed that the Delta variant was highly transmissible [10]. In addition, the Delta variant had been associated with multiple vaccine breakthrough infections [11].

The Delta variant is highly contagious, estimated to be more than two times as contagious as previous variants, and some data suggest that the Delta variant might cause more severe illness than other strains in unvaccinated persons [12]. Although infections with this variant happen in only a small proportion of fully vaccinated people, preliminary evidence suggests that fully vaccinated people who become infected with the Delta variant can spread the virus to others [13]. The low vaccination coverage in many communities is driving the current rapid and significant surge in cases associated with the Delta variant, which in turn would enhance the chances of the emergence of more concerning variants [12].

On 26 July 2021, a group of nearly 60 major medical organizations, including the American Medical Association, American Nursing Association, American Pharmacists Association, American College of Physicians, American College of Preventative Medicine, and American Public Health Association, advocated for all health care and long-term care employers to mandate that their employees be vaccinated against COVID-19 [14]. Vaccines are highly effective at preventing symptomatic disease, as shown by clinical trials and real-world evidence [3]. However, data on the effectiveness of these vaccines against this variant based on clinical outcomes are limited.

Knowledge of healthcare workers’ (HCWs) knowledge of COVID-19 variants has been evaluated in relation to the B.1.1.7 lineage; it was found that 97.3% were aware of its emergence, 78% believed that it caused more severe disease, and 50% thought that current COVID-19 vaccines were effective [15]. However, to date, no studies addressing HCWs’ knowledge and perception of the newly emerging Delta variant have been reported. In light of this, we aimed to investigate HCWs’ perceptions of the new Delta variant, their worries, and their acceptance of a vaccine booster.

## 2. Methods

### 2.1. Data Collection

This national cross-sectional survey was conducted in Saudi Arabia among HCWs between 9 and 14 August 2021. At the time of data collection, several countries had reported infections with the Delta variant of SARS-CoV-2. HCWs were invited to participate in this study using a convenience sampling technique through several healthcare providers’ social media platforms and email lists. Participants were surveyed regarding their perceptions of the Delta variant, interest in receiving a booster vaccination, and stress levels. The survey was a pilot-validated questionnaire sent through SurveyMonkey©, a platform that allows researchers to carry out surveys via the web. The survey was adapted from the one used in our previously published research, with modifications and additions related to the new Delta SARS-CoV-2 variant [15,16,17,18,19]. 

The questions asked related to the HCWs’ demographics (job category, age, sex, and work area), their previous exposure to COVID-19 patients, whether the HCW had previously been infected with COVID-19 themselves, whether they had traveled to a country with the Delta variant in the previous three months, the type of COVID-19 vaccine they had received, and their acceptance of a vaccine booster dose. We also assessed the following outcomes related to the Delta SARS-CoV-2 variant: knowledge (9 questions), perceptions, and travel worries. In addition, we assessed factors affecting the HCWs’ level of worry regarding international travel and their sources of information about SARS-CoV-2 variants. HCWs’ anxiety was also measured by asking them to self-rate their worry levels on a 5-item Likert scale, comparing their feelings of worry about the original COVID-19 strain and the Alpha and Delta variants.

In the first part of the survey, participants were informed of the purpose of the study and that their participation in this research was completely voluntary. The Institutional Review Board at the College of Medicine and King Saud University Medical City approved the study (approval #21/0650/IRB).

### 2.2. Statistical Analyses

Continuously measured variables were described using means and standard deviations, categorically measured variables were described using frequencies and percentages. Histograms and statistical Kolmogorov–Smirnov tests were used to assess the statistical normality of continuous measured variables. Multiple response dichotomy analyses were applied to multiple option questions such as HCWs’ sources of information. The HCWs’ knowledge of the Delta variant was described using the median and standard deviation of the total score achieved for the nine knowledge questions (all questions were given equal weight). The independent samples t-test and one-way ANOVA test were used to assess the statistical significance of the HCW’s mean perceived agreement with receiving a booster dose of the vaccine across the levels of their categorically measured variables, while Welch’s adjusted one-way ANOVA was used to correct for unequal variances for some of the tests. Pearson’s bivariate test of correlation was used to assess the correlations between metric variables. The chi-squared test of independence was used to assess the associations between categorically measured variables, and a paired samples t-test was used to compare HCWs’ worries about the various COVID-19 strains. Multivariate linear regression analysis was used to assess the associations between the HCWs’ characteristics, perceptions of the Delta variant, and willingness to receive a vaccine booster dose to prevent its spread. The SPSS IBM V21 (IBM, Armonk, NY, USA) program was used for the statistical data analysis and the EXCEL program (Microsoft Corporation, Redmond, WA, USA) was used for the creation of figures. The Alpha significance level was considered at the level of 0.050.

## 3. Results

A total of 1279 respondents (HCWs) completed the online survey. The majority were females (62%). Their age distribution is shown in Table 1. Regarding their clinical role, (41.7%) were nurses and (24.7%) were consultants. Their clinical assignment was evenly distributed across their institutes, apart from some clustering in the ICU (17.8%), general wards (28.4%), and (22.5%) Outpatient Department (OPD). The majority (69%) worked in tertiary hospitals. 

Most of the respondents had been in contact with COVID-19 patients (76.3%), while (23.2%) had personally been infected with it.

The surveyed HCWs reported high levels of traveling worry (mean 2.32/5 SD 1.1) and a minority (7.1%) had traveled to countries where the COVID-19 Delta variant had been recorded. Interestingly the highest perceived travel worry level was to the Original strain (3.14/5); followed by the Alpha variant (1.98/5) then the Delta variant (1.79/5). The HCWs reported significantly lower levels of travel worry attributed to the Delta variant compared to that reported for both the Alpha variant and the original strain (Table 2 and Table A1 in the Appendix A).

Table 3 shows the surveyed HCWs’ perceptions and practices to the different COVID-19 vaccines. Only (0.6%) had not received their first COVID-19 vaccine dose, while those who had been vaccinated were evenly distributed between receiving the Pfizer/BioNTech BNT162b2 or the AstraZeneca ChAdOx1nCoV-19 vaccines, which have both been authorized by the Saudi Food and Drug Administration (SFDA). At the time of the survey, 15.6% of the respondents had not received their second dose, though the majority (69.2%) had received their second dose with Pfizer/BioNTech BNT162b2 and the remainder had received the AstraZeneca ChAdOx1nCoV-19 vaccine.

Regarding their knowledge of the Delta variant, the majority (63.8%) admitted that they had limited knowledge about it, while 7.5% had no information about it at all. In contrast, 47.9% knew only limited information about the Delta-Plus variant, while 37.5% had no information about it at all (Table 3).

Assessing the respondents’ opinions (measured by a 5-point Likert scale) regarding their willingness to receive a third booster dose of COVID-19 vaccine to prevent the spread of the Delta variant and the efficacy of the currently available vaccines against it, their overall likelihood of accepting a booster dose was determined to be 3.67 out of 5. Their perception that the Pfizer vaccine would be effective against it was 3.71, while their perception of the effectiveness of using a mixture of Pfizer and AstraZeneca vaccines was 3.66. On the other hand, their expectation that the AstraZeneca vaccine would be effective against the Delta variant by itself was the lowest at 3.59.

A total of 708 (55.3%) of the respondents (with a mean likelihood score of 3.67 and SD of 0.94) stated that they would prefer to receive a booster dose of COVID-19 vaccine to help prevent the further spread of the Delta variant, 32.3% stated that they would prefer to receive the same type of vaccine that they had previously received, 31.5% stated that they would prefer to receive an mRNA vaccine that had been developed specifically for the Delta variant, and 16.8% stated that they would be comfortable with receiving any mRNA vaccine. The majority of the participants (77.8%) felt that HCWs should receive a booster dose, while only 1.6% believed that children should, and the majority of them believed that elderly people and patients with comorbidities should receive also receive a booster (details are shown in Table 3).

Table 4 highlights the expectations of the HCWs regarding the practices needed to combat the COVID-19 Delta variant. A total of 57% of the respondents believed that the Delta variant was not currently circulating in the KSA, while 40% thought that it was. In answer to a question concerning the participants’ opinions about the variant’s potential to cause another wave of COVID-19 in Saudi Arabia, 66% stated that they expected that it would cause another wave, while 5.2% disagreed with this, and 55.4% stated that they believed it would cause another lockdown only if there was another wave, while 12.7% disagreed with this. Interestingly, 84.2% of the surveyed HCWs agreed that universal masking should continue due to the emergence of COVID-19 mutations.

Table 5 shows the respondents’ scores for their knowledge about COVID-19 and its Delta variant using the 9 questions on the survey. Most of them knew that further COVID-19 mutations were expected (90.5%) and that the Delta variant was first described in India (79%), but only 53.8% of them knew that the new variant is as transmissible as chickenpox. A total of 78.4% of them knew that the variant causes more severe disease compared to the original strain, but 73% did not. Most of the HCWs did not know that the variant does not present with the same signs and symptoms as the original strain.

The majority (83.6%) of the HCWs erroneously believed that the appearance of mutant strains is a sign of herd immunity, and 83.9% did not know that mutant strains may cause negative PCR tests.

Most of the respondents thought that mixing vaccines would offer them more protection against the Delta variant (64%).

Table 6 explores the association between the surveyed HCWs’ knowledge score, their clinical role, and their sources of information. The respondents’ median knowledge score for the COVID-19 Delta variant was 55.6%; therefore, we split them into low score achievers (<55.6%) and high score achievers (>55.6%). The chi squared test of the independence of the association between respondents’ clinical roles and their average knowledge score showed significantly lower scores for technicians compared to the others (*p* = 0.012).

Nurses were significantly more inclined to use the World Health Organization (WHO) website as a source of information (*p* = 0.010) and were significantly more likely to use the hospital announcements than the other respondents (*p* = 0.022). In contrast, they were significantly less inclined to use the Ministry of Health (MOH)’s official statements than the others (*p* = 0.036). The use of the Saudi MOH and Saudi Centre of Disease Control (CDC) websites as sources of information did not differ significantly based on the respondents’ clinical roles. Consultants used the CDC website as a source of information more often than the others (*p* = 0.004). Social networks, which were the dominant source of information for all the surveyed HCWs, were used more often by the nurses than by the other respondents (*p* < 0.001).

Regarding scientific journals, consultants used them significantly more compared to other respondents (*p* < 0.001). The use of TV and news reports did not differ significantly between the respondents (*p* = 0.820). 

**Figure 1:**
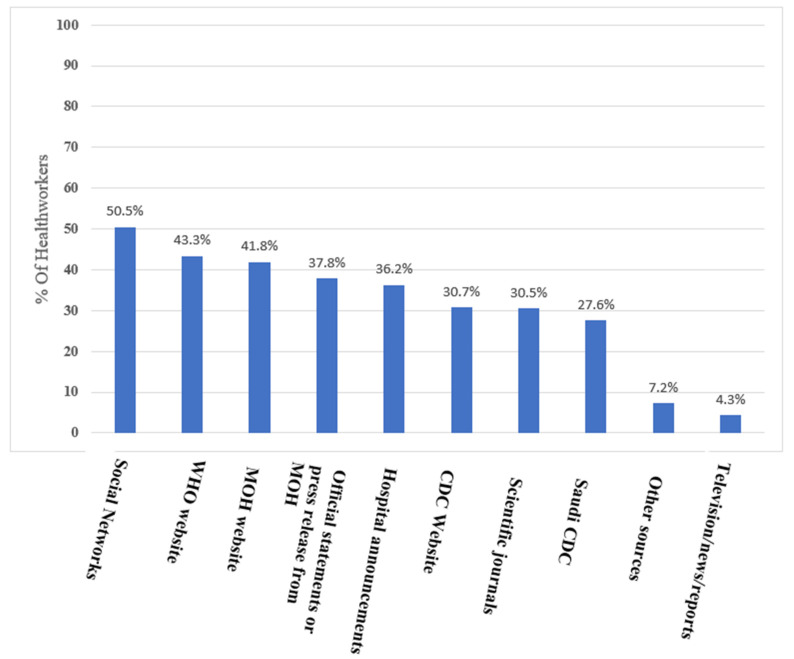
Health workers’ sources of Delta COVID-19 information.

Figure 1 shows the rate of utilization of the different sources of information based on the surveyed respondents’ responses. Social networks were the dominant source of information (50.5%), followed by the WHO website (43.3%) and the MOH website (41.8%), while the CDC website and scientific journals were used by 30.7% and 30.5%, respectively. 

The respondents’ perception that a booster dose is needed to prevent the spread of the Delta variant correlated positively and significantly with their belief in the effectiveness of the AstraZeneca vaccine against the Delta variant (r = 0.201 *p* < 0.010), their belief in the effectiveness of the Pfizer vaccine against it (r = 0.295 *p* < 0.010), and their belief in the effectiveness of mixing and matching vaccine types against the Delta variant; r = 0.295, *p* < 0.010. However, interestingly their perception of the need for a booster dose correlated significantly and positively but weakly with their knowledge score and their perceived level of worry about travel (Table 7). The respondents’ knowledge score relating to the Delta variant correlated positively and significantly with their worry about traveling abroad (r = 0.114, *p* < 0.010) but very weakly with their perceived level of worry about the Delta variant itself (r = 0.066, *p* < 0.05). Their knowledge score for the Delta variant converged statistically and positively with their belief of the effectiveness of the AstraZeneca vaccine, the Pfizer vaccine, and the mixing and matching of both vaccines against it, at *p* < 0.001 each.

Participants’ worries relating to traveling abroad correlated significantly and positively with their perceived level of worry from all the variants (the original COVID-19 strain, the Alpha variant, and the Delta variants), at *p* < 0.010 each. The respondents’ belief about the effectiveness of the AstraZeneca vaccine, the Pfizer vaccine, and mixing and matching both vaccines against the Delta variant all correlated significantly and positively with each other, at *p* < 0.010 (Table 7).

The multivariate linear regression analysis of the HCWs’ responses and characteristics revealed that their willingness to receive a booster dose of COVID-19 vaccine to prevent the spread of the Delta variant (Table 8) was significantly associated with being a Saudi national.

Receiving both doses of the COVID-19 vaccine regardless of their type was associated with a higher willingness to receive a booster dose. Considering the respondents’ high knowledge score for the Delta variant, high perception of the need for a lockdown if it causes an outbreak, and high perception of the need to comply with universal masking to prevent further mutation, all of these were associated with a high willingness to receive a booster dose to prevent the spread of the Delta variant.

On the other hand, there was no strong association between respondents’ expectations that the AstraZeneca vaccine might be effective against the Delta variant and their agreement to receive a third dose. Their expectations that the Pfizer vaccine or the mix and match approach currently being practiced (consisting of being vaccinated with one dose of Pfizer and receiving a second dose of AstraZeneca) were effective were both significantly associated with a higher willingness to receive a booster dose.

## 4. Discussion

In this first reported survey on the perceptions of the SARS-CoV-2 Delta variant worries and vaccine booster acceptability among HCWs in Saudi Arabia, a low level of knowledge and high level of worry about the delta variant, as well as a modest acceptance of the vaccine booster, were observed. Although the Saudi MOH recently announced that the Delta variant is circulating in the KSA after these data were collected, many HCWs anticipated that this variant would be encountered. While most of our study respondents (62.2%) were female, this is in line with other studies that showed a predominance of the female sex among frontline COVID-19 HCWs [15,20]. Among the estimated 49 million frontline HCWs in the E.U., around 76% are female [21].

In this survey, 23.2% of all the HCW respondents had previously been infected with COVID-19. In one study on the capital city, Riyadh, it was found that 12% of 1358 HCWs had tested positive for SARS-CoV-2 [22]. The source of SARS-CoV-2 infection among HCWs was mainly the community, with rates as high as 61.3% found in a study from Saudi Arabia and 25.5% in another study from Oman [22,23]. However, it is important to note that seroprevalence studies showed a low prevalence of 2.3% early in the pandemic, and this rate rose to 12.2% in another study [24,25]. It is interesting to note that the majority of HCWs were aware of the newly described Delta variant but had much less knowledge about the Delta-Plus variant, which could be explained by the media focusing on the Delta variant, previous study has shown Saudi students having satisfactory knowledge about the COVID-19 but poor knowledge about its mode of transmission and background of the disease highlighting the emphasis of further education and training especially for HCWs in our case about the dynamics of the disease rather than the media quality of knowledge [26,27,28,29]. The low percentage of professionals with a good knowledge of the variant may be related to the source of their information mainly being social networks; this also may be related to the professionals who were included, as 50.9% worked in general wards and OPD and 41.7% were nurses. The knowledge of HCWs about the emergence of the B.1.1.7 (Alpha) variant was reported to be 97.3%, while 78% and 50%, respectively, indicated that the alpha variant would cause more severe disease and that the current COVID-19 vaccines are effective against it [15]. Of the respondents in the current study, it was found that 63.8% had limited information about the Delta variant, which is much lower than the percentage cited in relation to the B.1.1.7 variant. 

With the emergence of the Delta variant, discussion about adding a booster dose of the COVID-19 vaccine has been increasing, with some countries already applying the 3rd dose booster for certain high-risk groups. In this questionnaire, 77.8% of the respondents agreed that a booster dose would be needed. The need for a booster dose was based on studies showing COVID-19 vaccines to be less effective against the Delta variant. In one study, two doses of the BNT162b2 vaccine were found to be 88% effective against the Delta variant and 93.7% effective against the Alpha variant, while the ChAdOx1 nCoV-19 vaccine was found to be 74.5% effective among persons with the Alpha variant and 67% among those with the Delta variant. Another study showed that the neutralizing antibodies generated by two doses were 3–5 fold lower than those generated by the Delta variant [8]. In addition, the viral loads of those infected with the Delta variant were similar among vaccinated and unvaccinated individuals [30]. In line with these scientific findings [3], 78.4% of the surveyed HCWs agreed that the Delta variant might cause worse disease than previous strains. However, it was interesting to note that 85.6% incorrectly agreed that the appearance of different viral strains signals herd immunity. The development of herd immunity is certainly a phenomenon that will be necessary to end this pandemic. Delta variants have emerged in multiple localities where herd immunity has not been achieved. The current data show that the Delta variant is more likely to cause disease among those who have received a single dose of the vaccine than among those who have had two doses [31]. The majority of the respondents agreed that the Delta variant is more transmissible than previous strains. Studies have shown that the transmissibility of the Delta variant is about 60% more than that of the original variant, with an estimated Ro of 5–8 [32]. 

The HCWs who were willing to receive a third booster dose were asked to select the type of vaccine they would prefer. The findings showed that 32.3% of them preferred to receive the same type of vaccine that they received for the first dose, while another 18.5% preferred it to be the same as the second vaccine dose type received, as indicated from the data from 200 HCWs who received a second dose of Pfizer/BioNTech BNT162b2 instead of their original first dose of AstraZeneca ChAdOx1nCoV-19. Both these vaccines were designed based on the original Wuhan SARS-CoV-2 Spike (S) protein [33], with their efficacy relying on stimulating appropriate antibody and T cell responses. Due to changes in the RBD region of the S protein from the Delta variant, the efficacy of both these vaccines may be diminished [34]. Serum from 25 individuals who received two doses of BNT162b2 and another 25 individuals who received two doses of ChAdOx1nCoV-19 geometric mean neutralization (GMT) titers against the Delta variant was reduced 2.7- and 2.6-fold, respectively, compared to the wild-type virus. In addition, there was a complete absence of GMT in 20 individuals who received only one dose of BNT162b2 [35].

In a test-negative case–control study from compared vaccination status in persons with symptomatic COVID-19 with vaccination status in persons who reported symptoms but had a negative test, both the BNT162b2 and ChAdOx1 nCoV-19 vaccine effectiveness were determined against symptomatic disease caused by the Delta variant compared to the Alpha variant. In total, 19,109 cases were included, 14,837 with the Alpha variant and 4272 with the Delta. In the pooled analysis for any of the two vaccines, effectiveness was lower after the first vaccine dose among persons with the Delta variant (30.7%; 95% CI, 25.2 to 35.7) than among those with the Alpha variant (48.7%; 95% CI, 45.5 to 51.7). Results for the first dose were similar for both vaccines, with an absolute difference in vaccine effectiveness against the Delta variant compared with the Alpha variant of 11.9 percentage points with the BNT162b2 and 18.7 percentage points with the ChAdOx1 nCoV-19 vaccines. In the pooled analysis for two doses for any of the vaccines, the effectiveness was 87.5% (95% CI, 85.1 to 89.5) with the Alpha variant and 79.6% (95% CI, 76.7 to 82.1) with the Delta variant. For BNT162b2, a slight difference in effectiveness between variants was observed after the second dose: 93.7% (95% CI, 91.6 to 95.3) with the Alpha variant and 88.0% (95% CI, 85.3 to 90.1) with the Delta variant. ChAdOx1 nCoV-19 was 74.5% (95% CI, 68.4 to 79.4) effective against Alpha and 67.0% (95% CI, 61.3 to 71.8) effective against Delta; however, the number of cases and the follow up period were insufficient to determine its effectiveness against hospitalization and death [3].

Around a third of the participants indicated that they would prefer to receive a new mRNA vaccine developed specifically for the Delta variant. Recently, modifications have been made for mRNA vaccines to target specific variants [36], although clinical trials are still lacking. Around 16% thought that it did not matter what type of booster the third dose should be. In an observational study that investigated the serological profiles of four healthy individuals who received two doses of the BNT162b2 vaccine, followed by a third booster dose with the Ad26.COV2.S vaccine, the third vaccine dose robustly increased titers beyond that of two vaccinations, and these elicited antibodies had neutralizing capability against all SARS-CoV-2 strains, except for one individual against B.1.351 in the latter assay [37]. 

Most participants thought that a booster dose would be recommended to various populations, including the elderly and immunocompromised individuals. On 18 August 2021, the U.S. Department of Health and Human Services (HHS) announced that the available data clearly show that protection against SARS-CoV-2 infection begins to decrease over time following the initial doses of vaccination and in association with the dominance of the Delta variant, evidence of reduced protection against mild and moderate disease has emerged. Based on their latest assessment, they concluded that the current protection against severe disease, hospitalization, and death could diminish in the months ahead, especially among those who are at higher risk or who were vaccinated during the earlier phases of the vaccination rollout. Hence, they concluded that a booster vaccine would be needed to maximize vaccine-induced protection and prolong its durability. Our data show the high awareness of HCWs of the latter conclusion, which would aid in a faster rollout of the booster dose among HCWs and their patients [38].

In the KSA, the Pfizer-BioNTech and Oxford-AstraZeneca vaccines were the first two to be approved by the SFDA. However, there has been growing concern about the level of protection that approved vaccines can provide, as well as the evasion mechanisms. In early May 2021, of 321 patients hospitalized with COVID-19 at a tertiary care center in Riyadh, 131 had the Delta variant [7]. A recent study from Qatar, a neighboring country to KSA, showed an accelerating decline in the effectiveness of BNT162b2 against all variants to 20% five to seven months after the second dose [39]. 

Most HCWs had had previous contact with COVID-19 patients, and 23.2% had been previously diagnosed with laboratory-confirmed COVID-19 themselves. This is a significant rise from previously published data in KSA of self-reporting the diagnosis of COVID-19 among HCWs, which was 10% overall [18]. In our study, 7.1% reported traveling internationally to countries with caseloads of the Delta variant, and international travel has been described as a risk factor for acquiring the Delta variant early in its emergence. A large cohort study from England on more than 8500 cases of the Delta variant showed that it was sequenced in 29.5% of international travelers vs. 10.1% of non-travelers [6].

Most of the HCWs agreed that the Delta variant could cause a new COVID-19 wave within Saudi Arabia, although the reported cases were still limited at the time of our study [7]. This could represent the knowledge of the HCWs of the rapidly evolution of the Delta variant globally, as the COVID-19 virus is becoming ‘fitter and faster’ [40]. The majority of the participants in our study also agreed that HCWs should maximize their compliance with universal precautionary measures considering the new COVID-19 mutations. This is particularly important as the Delta variant has a higher viral load and is hyper-transmissible, though most of the current understanding on SARS-CoV-2 predates the Delta variant, including epidemiological and prevention data, adhering to basic infection control measures with universal masking and social distancing is vital due to the emergence of this new threat.

The mental health impact of the COVID-19 pandemic is unfolding, with more reports about this phenomenon being published. For example, a recent meta-analysis estimated that at least one in five healthcare professionals reported symptoms of depression and anxiety [41]. In addition, frontline HCWs who have daily direct exposure to patients with COVID-19 are considered to be at a higher risk of developing mental health problems [42,43,44]. Regardless of the relevance of clinical research, HCWs, whether frontline or non-frontline, are susceptible to infection and may experience substantial psychological stress [45]. However, the arrival of the Delta variant has also brought new awareness about the potential of COVID-19 to evolve and perhaps linger in the upcoming months and years [46]. This unique insight might explain why HCWs’ knowledge level about the Delta variant was higher than that for the other variants.

Similarly, high worry levels due to this new mutation and strong beliefs in the vaccines’ effectiveness might help to develop a positive perception of the booster dose to prevent its spread, as observed in our sample. Furthermore, HCWs’ knowledge of the Delta variant and its positive and significant association with worry about international travel was not surprising, as this finding has been reported before with other variants [15]. However, in our study, more than half of the survey respondents were non-Saudi expatriates, and this worry reflects how much they anticipate that this new variant might restrict their ability to travel. 

The uptake of the Pfizer vaccine dominated for the second dose in approximately 70% of the surveyed HCWs. This could reflect the higher availability of the Pfizer vaccine, the limited supply of AstraZeneca, the preferences of HCWs, as well as the approval of the mix and match COVID-19 vaccination regimen. The prime reason for supporting the mix and match of Pfizer/AstraZeneca in different countries is the potential benefits of triggering a higher immune response, better efficacy, and better effectiveness. It could also lower some safety concerns encountered with some vaccines [47], vaccine uptake hesitancy is an issue that has to be taken with high consideration when vaccine choice would depend on the availability rather than personal desire [48].

The reduction in the uptake of the second dose from February to May was apparently due to the MOH’s policy of administering the first dose widely and delaying the second dose for all except for a specific group of high-risk population, including persons who are more than 60 years and those with chronic diseases. The resumption of the second dose for HCWs was issued on June 25; however, the peak uptake was remarkably higher in July, a time just before the vaccine mandate in the country was put in place to start on August 1. This mandate requires COVID-19 vaccination for entry to all work premises, including healthcare institutions and public facilities. 

COVID-19 vaccine mandates are currently sparking discussions in many countries, including about ethical and legal considerations [49]. Before the set date of mandate, the MOH had launched intensive motivational vaccination programs, organized multisite vaccination units, stressed personal responsibility, and coordinated with both the public and private sector to encourage and promote vaccine uptake.

Our findings about the circulation of information about the new COVID-19 Delta variant among HCWs in Saudi Arabia were thought-provoking. Our study shows that the top source of information relating to this new variant was social media networks, according to 50.5% of the HCWs, which is comparable to the top source of information reported in a study published about the B.1.1.7 variant and healthcare workers’ travel worries in Saudi Arabia, which showed a result of 67%; this reflects the need for strict content monitoring and the regulation of social media [15]. The second most accessed source was the Ministry of Health website, followed by the Centre of Disease Control and Prevention (CDC) website, despite the fact that such websites have developed easily reached information tracking systems for HCWs [50,51]. The role of scientific journal articles was a concern, as only 30.5% relied on these for information, and these were mainly consultant physicians. This is despite the fact that many journals have fast-tracked publications related to new COVID-19 variants and made them open access. This phenomenon was also observed in an Indian study that tested the “infodemic” of COVID-19 among healthcare professionals [52]. The poor use of medical journals as the primary source of information about COVID-19 among HCWs is not yet well understood and warrants further research.

In our study, we noticed that the overall Delta variant knowledge score differed significantly between HCWs; those working in technical jobs were found to be significantly less likely to have high overall knowledge about the Delta variant. Therefore, training all HCWs about the new COVID-19 variants and how to gather information from more trusted and scientific channels is necessary in order to avoid misinformation, as advocated by the Saudi Ministry of Health (MOH) and other healthcare authorities [53,54].

The HCWs’ COVID-19 Delta variant knowledge score was significantly associated with their level of worry about traveling abroad; their level of worry about traveling was correlated significantly and individually with their perceived level of worry about the original strain, the Alpha variant, and the Delta variant. Their Delta variant knowledge score significantly correlated with their belief in the effectiveness of the AstraZeneca and Pfizer vaccines and the mix and match approach against the Delta variant. Additionally, their beliefs about both of the mentioned vaccine strategies were significantly correlated with each other. Such associations between the knowledge, the worry level, and multiple vaccination strategies reflect the level of anxiety HCWs are experiencing, their hopes about stopping the pandemic, and their feelings about relying on vaccines to achieve that [55].

Our results did not find any significant association between the HCWs’ previous personal infection with COVID-19 and their willingness to receive a booster dose to prevent the spread of the Delta variant. Such an attitude might not be a safe strategy, as the recent recommendation from the CDC is that patients who have been infected with COVID-19 should receive complete vaccination, the same as people who have not been infected; therefore, a third booster dose might be recommended, as the previous infection might not offer any added protection [56].

HCWs who had received two doses of vaccine had a significant level of willingness to receive a booster dose, which might reflect their awareness that their immunity level may be waning, especially since many of them had received their first dose 6–8 months prior [57]. A Polish study showed that 96% of HCWs hoped vaccines would stop the pandemic, and that the majority did not mind experiencing minor adverse effects. Similar to our study, such perceptions might strongly encourage HCWs to be willing to receive a booster dose [42].

Surprisingly, the knowledge scores relating to the Delta variant in our cohort did not converge with their willingness to receive a booster dose, which might be related to the overall low mean and median scores for knowledge. Added to that, the HCWs’ perceptions about whether the Delta variant was already circulating in Saudi Arabia did not correlate with their willingness to receive a booster dose, which could be explained by the fact that only the minority of them knew that it was.

It is known that mRNA vaccines induce a robust T cell response to emerging viral variants, and that the mix and match vaccine strategy offers robust immunogenicity and tolerable reactogenicity. The HCWs who were aware of such information showed a higher willingness to receive a third booster dose [58,59].

### Study Limitations and Strengths

Our research is subject to the limitation of cross-sectional studies, such as sampling as it was done with convenient sampling technique not non-probability technique which is more ideal, and that is because the latter is not easily feasible due to difficulty of having fair access to all the HCWs in the country, adding to being costly and time and labor consuming, response, and recall biases. While this study is among the first in our region to explore perceptions and worries among HCWs relating to the new SARS-CoV-2 Delta variant, their experiences and perceptions are likely to change, as the MOH announced on 15 August 2021, after our data collection was complete, that the Delta variant is circulating in Saudi Arabia [60]. As the percentage of the country’s HCWs who received the survey was not known, we were unable to calculate the response rate. Moreover, HCWs’ perceptions may differ from one setting to another. The factors of whether HCWs have received both COVID-19 vaccine doses themselves and what vaccine type they received may alter their level of worry about the new variants; future research could explore this further.

## 5. Conclusions

This was the first national survey conducted in Saudi Arabia addressing both the perception of the SARS-CoV-2 Delta variant and vaccine booster dose acceptance among HCWs. During the early time of the Delta wave, a high percentage of HCWs were aware of the variant, but most of them had limited overall knowledge about its contagiousness, presentation, being different than the original strain of SARS-CoV-2, its ability to cause false-negative PCR and the high efficacy of mixing mRNA and the other viral vector COVID-19 vaccines against the mutant variants. The HCWs exhibited high worry levels and a modest acceptance of receiving a vaccine booster dose. These results could guide public health officials to scale up educational efforts to disseminate reliable information on the different variants and provide recommendations about receiving a vaccine booster. Further research on methods to alleviate HCWs’ worries about emerging variants is warranted. 

## Figures and Tables

**Table 1:** Descriptive analysis of respondents’ sociodemographic and job characteristics. *n* = 1279.

Parameter	Frequency	Percentage
Sex		
Female	795	62.2
Male	484	37.8
**Age (years), mean (SD)**	38.56 (9.17)	
**Age group**		
20–30 years	253	19.8
31–40 years	581	45.4
41–50 years	280	21.9
>50 years	165	12.9
**Nationality**		
Saudi	578	45.2
Expatriate	701	54.8
**Clinical Role**		
Consultant	316	24.7
Assistant consultant/fellow	93	7.3
Resident/registrar/physician in training	244	19.1
Nurse	533	41.7
Others: pharmacist, lab technician (radiology and anesthesia)	92	7.2
**Health institution working area**		
ICU	228	17.8
ER	126	9.9
OR	79	6.2
Isolation ward	48	3.8
General ward	363	28.4
OPD	287	22.5
Non-clinical area	66	5.2
Hospital auxiliary units: radiology, sleep lab, pharmacy	81	6.3
**Hospital setting**		
Private	254	19.9
Public/governmental	448	35
University hospital	577	45.1
**Hospital type of practice**		
Primary healthcare center	192	15
Secondary-care hospital	204	15.9
Tertiary hospital	883	69
**Travel during the last three months to a country where the Delta variant has been recorded**		
No	1187	92.9
Yes	91	7.1
**Have you been in contact with people infected with COVID-19?**		
Never	303	23.7
Yes, with patients or family members	975	76.3
**Have you previously been diagnosed with PCR-positive COVID-19?**		
No	982	76.8
Yes	296	23.2

**Table 2:** Healthcare workers’ (HCWs’) perceived travel worry attributed to COVID-19 and its strains.

HCWs’ Perceived Travel Worry Level. Mean (SD)	2.32 (1.1)
**HCWs’ perceived worry level relating to COVID-19 strains**	
The original COVID-19 strain. Mean (SD)	3.14 (1.12)
The Alpha variant. Mean (SD)	1.98 (1.1)
The Delta variant. Mean (SD)	1.79 (1.00)

**Table 3:** Descriptive analysis of the respondents’ perceptions and practices relating to the COVID-19 vaccine and their perceptions and practices relating to the Delta variant.

HCW Practices/Perception	Frequency	Percentage
**What vaccine type did you take for your first dose of COVID-19 vaccine?**		
Pfizer	683	53.4
AstraZeneca	587	45.9
Not received	8	0.6
**What was the type of the second dose of COVID-19 vaccine you took?**		
Pfizer	884	69.2
AstraZeneca	194	15.2
Not received Yet	200	15.6
**Have you heard about/do you know about the Delta COVID-19 viral mutant?**		
Yes: I know a lot about this topic	367	28.7
Yes: I know limited information	815	63.8
No	96	7.5
**Have you heard about/do you know about the New Delta-Plus COVID-19 viral mutant?**		
Yes: I know a lot about this topic	187	14.6
Yes: I know limited information	612	47.9
No	479	37.5
**The AstraZeneca COVID-19 vaccine is effective against the Delta variant, mean (S.D.) Likert agreement score**	3.59 (0.76)	
**The Pfizer COVID-19 vaccine is effective against the Delta variant, mean (S.D.) Likert agreement score**	3.71 (0.76)	
**Mixing and matching one AstraZeneca vaccine and one Pfizer vaccine is effective against the Delta variant, mean (S.D.) Likert agreement score**	3.66 (0.80)	
**To help prevent the further spread of the Delta variant, a third booster dose of the COVID-19 vaccine will likely be needed, mean (S.D.) Likert agreement score**	3.67 (0.94)	
**What type of vaccine would you prefer to receive for the third booster COVID-19 vaccine? *n* = 708**		
Same as the first vaccine type	229	32.3
Same as the second vaccine type	131	18.5
A new mRNA vaccine that is developed to cover the Delta variant better	223	31.5
Another vaccine, not mRNA type	6	0.8
It does NOT matter; I would be okay with receiving any mRNA vaccine as a third booster	119	16.8
**In your opinion, who should receive a third booster COVID-19 vaccine dose?**		
Elderly people	546	77.1
Patients with diabetes	466	65.8
Patients with cardiovascular disease such as hypertension	442	62.4
Patients with chronic renal disease	442	62.4
Patients with immune deficiency	488	68.9
Healthcare workers	551	77.8
Obesity	255	36
All people and JCW travel jobs	58	8.2
Children	11	1.6

**Table 4:** HCWs’ expectations about the practices needed to combat the COVID-19 Delta variant.

HCW Practices/Perceptions	Frequency	Percentage
**To the best of your knowledge, is the Delta variant currently circulating in the KSA**		
Unsure	39	3.1
Yes	511	40
No	728	57
**This Delta variant has the potential to cause a new COVID-19 wave in the KSA**		
Agree	844	66
Neither agree nor disagree	368	28.8
Disagree	66	5.2
**A second national lockdown may be implemented if a Delta variant outbreak occurs**		
Agree	708	55.4
Neither agree nor disagree	408	31.9
Disagree	162	12.7
**Due to the new COVID-19 mutations, healthcare workers should continue universal masking**		
Agree	1076	84.2
Neither agree or disagree	190	14.9
Disagree	12	0.9

**Table 5:** Respondents’ knowledge score for the Delta COVID-19 variant.

Respondents’ Knowledge Statement (TRUE or FALSE) *	Incorrect Answer (%)	Correct Answer (%)
This COVID-19 Delta variant was first described in India (TRUE)	268 (21)	1010 (79)
The Delta variant is as transmissible as chickenpox (TRUE)	591 (46.2)	687 (53.8)
Mixing and matching the COVID-19 vaccine offers more protection against the Delta variant (TRUE)	460 (36)	818 (64)
The signs and symptoms of the Delta strain of COVID-19 are the same as those of the original strain (FALSE)	933 (73)	345 (27)
The COVID-19 mutations are expected (TRUE)	122 (9.5)	1156 (90.5)
The Delta variant causes more severe disease than the original strain (TRUE)	276 (21.6)	1002 (78.4)
The appearance of mutagenic viruses is a sign that herd immunity is occurring (FALSE)	1068 (83.6)	210 (16.4)
The mutation may cause a false negative PCR result (TRUE)	1072 (83.9)	206 (16.1)
Treatment for the disease caused by the mutation is similar to the protocol used for COVID-19 treatment (TRUE).	423 (33.1)	855 (66.9)

* The best correct answers are displayed in brackets ( ).

**Table 6:** Respondents’ Delta variant knowledge scores and sources based on their clinical role.

Clinical Role No. (%) Knowledge Parameter	Consultant	Assistant Consultant/Fellow	Resident/Registrar/ Physician in Training	Nurses	Technicians	*p*-Value
**HCWs’ Delta variant knowledge score**						
Low score (≤55.6%)	177 (56)	55 (59.1)	163 (66.8)	342 (64.2)	67 (72.8)	0.012
High score (>55.6%)	139 (44)	38 (40.9)	81 (33.2)	191 (35.8)	25 (27.2)	
**Source of information**						
Hospital announcements (e.g., roll-ups or newsletters)	107 (33.9)	23 (24.7)	68 (27.9)	202 (37.9)	33 (35.9)	0.022
Official statements or press release from MOH (e.g., through SMS or newspapers)	123 (38.9)	40 (43)	90 (36.9)	163 (30.6)	36 (39.1)	0.036
MOH website	111 (35.1)	33 (35.5)	90 (36.9)	225 (42.2)	41 (44.6)	0.169
WHO website	122 (38.6)	26 (28)	91 (37.3)	243 (45.6)	36 (39.1)	0.010
CDC Website	117 (37)	26 (28)	68 (27.9)	131 (24.6)	25 (27.2)	0.004
Saudi CDC	96 (30.4)	23 (24.7)	68 (27.9)	120 (22.5)	22 (23.9)	0.123
Social Networks (such as YouTube, Facebook, Twitter, WhatsApp)	104 (32.9)	36 (38.7)	98 (40.2)	326 (61.2)	39 (42.4)	<0.001
Scientific journals	151 (47.8)	32 (34.4)	69 (28.3)	87 (16.3)	25 (27.2)	<0.001
Other sources	17 (5.4)	5 (5.4)	10 (4.1)	53 (9.9)	1 (1.1)	0.002
Television/news/reports	13 (4.1)	5 (5.4)	7 (2.9)	24 (4.5)	4 (4.3)	0.820

**Table 7:** Bivariate Pearson’s correlations between the respondents’ measured knowledge, worry level, need for a third booster dose, and perception of the effectiveness of different vaccines.

Parameters	HCWs’ Perception Third Dose is Needed to Prevent the Spread of the Delta Variant	Knowledge of Delta Variant	Travel Worry Level	COVID-19 Original Strain Worry Level	Alpha Strain Worry Level	Delta Strain Worry Level	AstraZeneca Vaccine Effective Against Delta	Pfizer Vaccine Effective Against Delta
Knowledge of Delta variant	0.143 **							
Travel worry level	0.048	0.114 **						
COVID-19 original strain worry level	0.049	−0.077 **	0.629 **					
Alpha strain worry level	0.012	−0.128 **	0.570 **	0.722 **				
Delta strain worry level	0.072 *	0.066 *	0.696 **	0.677 **	0.679 **			
AstraZeneca vaccine effective against Delta	0.201 **	0.172 **	−0.029	−0.055 *	−0.081 **	−0.073 **		
Pfizer vaccine effective against Delta	0.295 **	0.213 **	−0.010	−0.042	−0.091 **	−0.036	0.649 **	
Vaccine mix/match effective against Delta	0.295 **	0.259 **	0.017	−0.048	−0.087 **	−0.012	0.565 **	0.579 **

** Correlation is significant at the 0.01 level (2-tailed). * Correlation is significant at the 0.05 level (2-tailed).

**Table 8:** Multivariate linear regression analysis of the respondent’s willingness to receive a COVID-19 vaccine booster dose.

Variable	Unstandardized Beta Coefficients	95.0% C.l. for Beta Coefficient		*p*-Value
		Lower Bound	Upper Bound	
Sex = Male	0.089	−0.019	0.196	0.106
Age (years)	−0.002	−0.008	0.003	0.450
Nationality = Saudi	−0.117	−0.220	−0.013	0.027
Previous exposure to people infected with COVID-19	0.077	−0.038	0.193	0.189
To the best of your knowledge, is the Delta variant currently circulating in KSA	−0.081	−0.172	0.010	0.081
Overall knowledge of Delta COVID-19 mutation	0.006	−0.029	0.042	0.723
Had taken first and second COVID-19 vaccine doses	0.182	0.048	0.316	0.008
Delta variant has the potential to cause a new wave in the KSA	0.073	−0.018	0.163	0.116
A second national lockdown may be implemented if a Delta variant outbreak occurs	0.124	0.051	0.198	0.001
Due to the new COVID-19 mutations, HCWs should continue universal masking	0.141	0.006	0.277	0.041
Awareness about the Delta variant	0.097	0.007	0.186	0.034
The AstraZeneca vaccine is effective against the Delta variant	−0.059	−0.147	0.029	0.188
The Pfizer vaccine is effective against the Delta variant	0.224	0.134	0.314	<0.001
Vaccine mixing/matching is effective against the Delta variant	0.220	0.141	0.298	<0.001

Dependent variable = respondents’ willingness to receive a 3rd COVID-19 vaccination dose. Significant *p* value is < 0.05.

**Figure A1:**
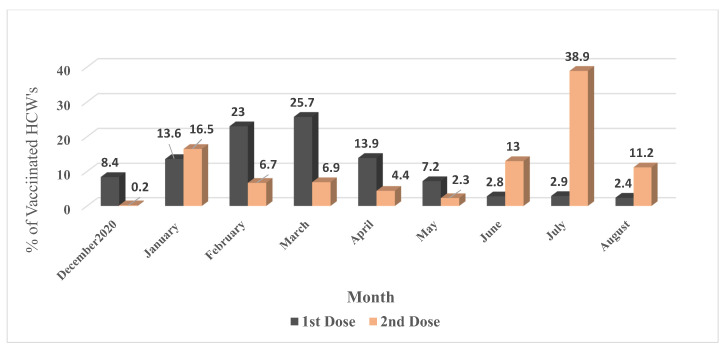
Monthly distribution of the HCWS vaccination of COVID-19 according to the responses.

## Data Availability

The data presented in this study are available upon reasonable request from the corresponding author.

## Abbreviations

CDC	Centers for Disease Control and Prevention.
COVID-19	coronavirus disease 2019.
MOH	Ministry of Health
SARS-CoV-2	severe acute respiratory syndrome coronavirus 2.
WHO	World Health Organization.

## Appendix A

Appendix AFigure A1 shows the respondents’ monthly rate of COVID-19 vaccine uptake. The vaccination campaign started 17 December 2020, in KSA; as evident from the graph, the vaccine uptake increased steadily since December 2020 for the first dose during the first four months, then the second dose steadily over subsequent months.

**Table A1:** Bivariate paired samples t-test comparing HCW’s worries from various COVID-19 strains.

Variables	t-Value/df	*p*-Value
Worry from Original strain vs. Alpha strain	8.52/1277	<0.001
Worry from alpha strain vs. Delta	1277/14.31	<0.001
Worry from Delta strain vs. Original strain	1277/22.62	<0.001
